# Alterations in corticospinal excitability in individuals with ankle sprains: a systematic review of transcranial magnetic stimulation studies

**DOI:** 10.3389/fphys.2026.1951700

**Published:** 2026-09-21

**Authors:** Yanli Chen, Mengyu Li, Songlin Xiao

**Affiliations:** School of Sports Medicine and Health, Chengdu Sport University, Chengdu, China

**Keywords:** acute lateral ankle sprain, chronic ankle instability, corticospinal excitability, motor evoked potential, transcranial magnetic stimulation

## Abstract

**Objective:**

This study aimed to systematically review the literature on corticospinal excitability in individuals with ankle sprains.

**Methods:**

This systematic review adhered to the guidelines established by the Preferred Reporting Items for Systematic Reviews and Meta-Analyses. A systematic search was performed on PubMed, SPORTDiscus, and Web of Science. Studies were included according to the Participants, Intervention, Comparison, Outcomes, and Setting inclusion criteria.

**Results:**

The electronic search yielded 233 studies, of which eleven studies were included after careful screening. Eight of the nine studies consistently reported reduced corticospinal excitability of several lower limb muscles in chronic ankle instability (CAI). Two studies identified an elevated motor evoked potential in cases of acute lateral ankle sprain.

**Conclusions:**

CAI exhibits lower corticospinal excitability and high cortical inhibitory activity. Individuals with acute ankle sprain exhibit increased corticospinal excitability, which be presented cautiously as a preliminary finding requiring further confirmation. This review offers a theoretical basis for revealing potential changes in spinal cord segmental activities after ankle sprains, suggesting that altered corticospinal excitability may associate with impaired sensorimotor function and represents a potential target for future rehabilitation research.

## 1 Introduction

Chronic ankle instability (CAI) is a prevalent chronic musculoskeletal injury of the lower extremities, with up to 70% of individuals experiencing persistent ‘giving way’ and recurrent sprains of the ankle joint after the initial acute ankle sprain, including medial sprains, syndesmotic ankle sprains (high ankle sprains), and lateral sprain (the most common) (Yeung et al., 1994; Doherty et al., 2014). CAI is associated with impaired postural control, decreased muscle strength, and weakened neuromuscular control (Hiller et al., 2011; McLeod et al., 2015). Persistent functional impairment can even lead to more severe injuries, such as osteoarthritis and ligament rupture (McLeod et al., 2015; Kosik et al., 2017; Kim et al., 2025a). CAI may result in increased rehabilitation costs and reductions in physical activity participation, potentially affecting health levels and quality of life (Kosik et al., 2017). Therefore, a thorough understanding of the underlying mechanisms of CAI is of considerable theoretical significance for preventing re-injury and designing effective rehabilitation strategies.

Recent studies have focused on the peripheral musculoskeletal system to explore the possible causes of ankle sprains, mainly including decreased muscle strength, reduced proprioception, neuromuscular control disorders, slow reaction time, and impaired balance control (Thompson et al., 2018; Nozu et al., 2021; Xue et al., 2023a). However, the regulation of ankle movement is governed by the cerebral nervous system (including the central and spinal cord components). Peripheral receptors (sensory receptors) collect the information and transmit it via the afferent nerves to the spinal cord segments and then ascend to the cerebral cortex. After integration and processing, they form relatively accurate movement instructions that are relayed to the muscles to meet various functional requirements of the ankle, such as maintaining balance and stability (Gilbert and Sigman, 2007). After ankle sprains, abnormalities in the above regulatory pathways may occur, leading to diminished information transmission to certain segments and a corresponding decrease in activity, which alters movement patterns and predisposes the individual to repeated sprains, which eventually develop into CAI (Needle et al., 2017). Therefore, in the injury mechanism of CAI, a crucial factor to consider is the potential changes in the cerebral nervous system. Notably, a few systematic reviews have reported on this topic, including brain neuroplasticity and spinal and corticospinal excitability related to CAI and ALAS in functional magnetic resonance imaging, electroencephalography, and transcranial magnetic stimulation (TMS) studies (Kim et al., 2019; Suttmiller and McCann, 2020; Maricot et al., 2023). However, these studies were mainly conducted five years ago or covered a rather broad range of topics. We thus further focus on TMS studies to determine the alterations in activity between spinal segments and the brain, which novelly summarize the corticospinal excitability in individuals with ALAS and CAI.

Transcranial magnetic stimulation (TMS) can evaluate corticospinal excitability by measuring motor evoked potential (MEP), cortical silent period (CSP), resting motor threshold (RMT), and latency. Previous studies have shown that the activity of spinal cord segment-related reflex circuits is affected following damage to the peripheral ligaments after ankle sprains. For example, the MEP of the fibularis longus (FL) decreases, which may significantly affect the prompt control of movement tasks and inevitably associated with recurrent sprains (McLeod et al., 2015; Kim et al., 2019; Nanbancha et al., 2019). Meanwhile, some studies observed minimal changes in the neurological activities of CAI individuals, such as no significant changes in the vastus medialis MEP (McLeod et al., 2015). Furthermore, some studies found that the MEP of the FL in individuals with early ALAS is high (Kim et al., 2025a, Kim et al., 2025b). Given the inconsistency of key factors, such as target muscles and injury duration, in existing research protocols, which results in significant variability in outcomes, researchers should systematically summarize the alterations in the corticospinal excitability in individuals with ankle sprains.

Therefore, this study aimed to systematically review the literature regarding corticospinal excitability in individuals with ankle sprains, including ALAS and CAI. Identifying the relationship between corticospinal excitability and peripheral foot–ankle sensorimotor function will guide future investigations in designing rehabilitation protocols for acute or recurrent ankle sprains.

## 2 Methods

This review was conducted in accordance with the guidelines of the Preferred Reporting Items for Systematic reviews and Meta-Analyses (Cumpston et al., 2019; Page et al., 2021).

### 2.1 Search strategy

According to the requirements of the database, we used the Boolean operators “AND” and “OR” to combine the keywords [“neural excitability” OR “supraspinal excitability” OR ”cortic* excitability” OR “motor neuron pool excitability” OR “motor evoked potential” OR “motor threshold” OR “transcranial magnetic stimulation” OR “spinal excitability” OR “cortical silent period” OR “corticospinal silent period” OR “corticospinal activity”] AND [“chronic ankle instability” OR “CAI” OR “acute lateral ankle sprains” OR “ALAS” OR “ankle instability” OR “ankle sprain” OR “functional ankle instability” OR “copper” OR “joint instability” OR “unstable ankle” OR “medial sprains”] for searches in the Web of Science, PubMed, and SPORTDiscus databases. The search period was from the establishment of the database to January 1, 2026, ensuring the inclusion of the latest research. Moreover, a manual review of the relevant references from the included studies was conducted to prevent the omission of relevant literature. All results from the search were imported into the EndNote reference manager (EndNote X9, USA, Stanford) to consolidate them and automatically detect duplicate records.

### 2.2 Selection criteria

Two researchers independently evaluated the results of the search and resolved differences through discussion (YL and MY). The abstracts and full texts of relevant studies were read thoroughly, and only those that met the eligibility criteria were selected. The researchers further confirmed the selected studies and discussed possible disagreements; if any disagreement persisted, then a third researcher was consulted to determine the results (SL).

#### 2.2.1 Inclusion criteria

Selection criteria were defined based on the Population, Intervention, Comparison, and Outcome framework: 1) The participants were individuals with ankle sprain (i.e., ALAS and CAI). The criteria were consistent with the recommendations of the International Ankle Consortium, including (I) at least 1 significant ankle sprain that occurred at least 12 months ago and resulted in an interruption of at least 1 day of desired physical activity, (II) the absence of any ankle injury in the past 3 months and (III) at least 2 episodes of ‘giving way’ in the past 6 months, (IV) self-reported ankle instability confirmed by a validated questionnaire (Gribble et al., 2014); 2) The control group comprised healthy controls (HCs) or copers; 3) The primary outcomes were evaluated using transcranial magnetic stimulation, mainly including MEP, CSP, and RMT (**Table 1**).

**Table 1:** Definition of the main variables measured by TMS.

Variables	Definition and meaning
Motor evoked potential (MEP)	The overall reaction of a peripheral muscle, measured by electromyography (EMG), that is induced via TMS of the contralateral motor cortex. A higher MEP indicates greater corticospinal excitability (Terada et al., 2022).
Cortical silent period (CSP)	A brief period of electrical silence in the surface EMG activity following a single-pulse TMS-evoked MEP. A longer CSP indicates greater corticospinal inhibition (Terada et al., 2022).
Resting motor threshold (RMT)	The minimal intensity of TMS required to consistently evoke a reliable MEP in a fully relaxed target muscle. A higher RMT indicates lower corticospinal excitability (Terada et al., 2022).
Active motor threshold (AMT)	The lowest stimulator intensity required to elicit at least four of eight MEP with peak-to-peak amplitudes that exceeded the MEP threshold. A higher AMT indicates lower corticospinal excitability (Groppa et al., 2012).
Latency	The duration of the neural signal transmissibility from the motor cortex to the targeted muscle. A higher latency indicates decreased conduction velocity in motor pathways (Nanbancha et al., 2019).

#### 2.2.2 Exclusion criteria

Republished literature, grey literature, reviews, conference proceedings, and case reports were excluded. Animal studies and non-English studies were also omitted.

### 2.3 Data extraction

Three reviewers performed data extraction. The original data from the included studies were summarized in Microsoft Excel (Microsoft Corporation, Redmond, WA, USA). The main information extracted from the data included the following: 1) basic information of the literature: authors and publication year; 2) participant demographics: the sample size, gender, and age of the ankle sprain and control groups; 3) main parameters of TMS testing: intensity of magnetic stimulus; 4) primary outcomes of TMS testing: MEP, AMT, CSP, and RMT; and 5) main findings: differences in primary outcomes between individuals with ankle sprains and the controls (**Table 1**). Besides, we also extracted the effect sizes (ES) for the main findings. When ES was not reported, we used R to calculate ES from the reported sample sizes, means, and standard deviations, and then used GraphPad Prism to present ES and 95% confidence intervals (CIs) in a forest plot without pooling (**Supplementary Figure 1**).

### 2.4 Quality and risk of bias assessment

Two researchers independently completed the quality and risk of bias assessment and resolved differences through discussion (YL and MY). If any disagreement persisted, then a third researcher was consulted to determine the results (SL). A modified Newcastle-Ottawa Quality Assessment Scale was used to assess the quality of the included cross-sectional studies (**Supplementary Table 2**) (Wells et al, 2026). Besides, the revised JBI critical appraisal tool for risk of bias assessment of analytical cross-sectional studies was used (**Supplementary Table 3**) (Barker et al., 2026).

## 3 Results

### 3.1 Search results

A rigorous search strategy yielded a total of 233 records: 100 from Web of Science, 55 from PubMed, 76 from SPORTDiscus, and 2 from reference list searches. After removing duplicate studies using EndNote X9, 126 studies were obtained. Following the screening of titles and abstracts, 29 studies were selected. Eleven studies were finally included after full-text screening. **Figure 1** illustrates the study selection process.

**Figure 1:**
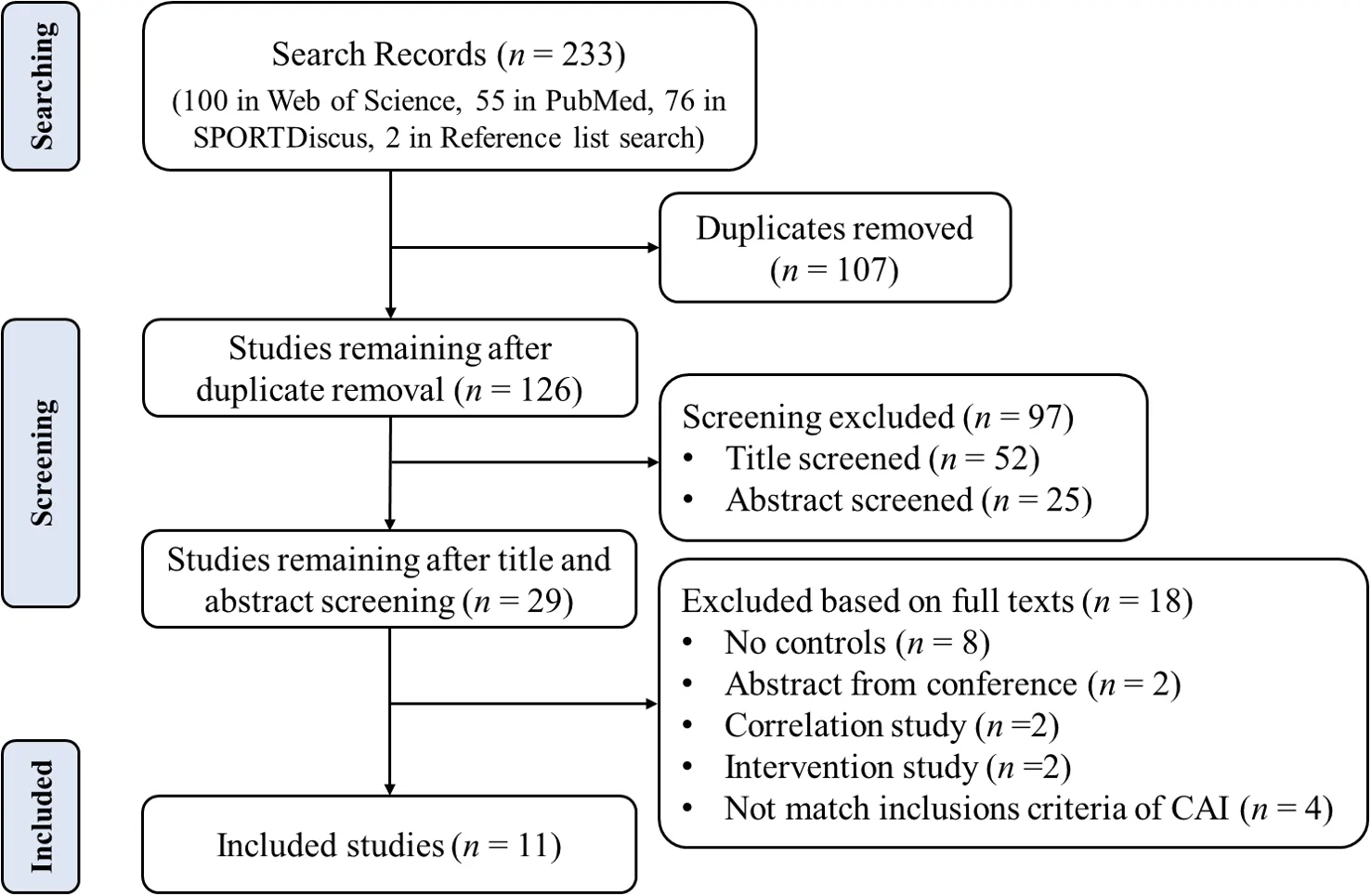
Flowchart of literature search and screening.

### 3.2 Risk-of-bias assessment

The quality of cross-sectional studies as assessed by NOS was high, with a median NOS score of 7/9 points (**Supplementary Table 2**). Ten eligible studies scored 7 out of total 9 points, while one study scored 8 out of 9 points. Besides, for the risk of bias evaluation, all studies exhibited a low risk, including Bias related to selection and allocation, bias related to classification of the exposure, bias related to assessment, detection, and measurement of the outcome, statistical conclusion validity, and comprehensiveness of reporting. Five studies did not identify the confounding factors and not deal with these confounding factors (Needle et al., 2013; Terada et al., 2016; Kosik et al., 2017; Thompson et al., 2020; Futatsubashi and Sekiguchi, 2024).

### 3.3 Basic characteristics of the included studies

This review included 11 studies involving 172 individuals with CAI, 27 individuals with ALAS, 33 copers, and 159 HCs. All included studies conducted a cross-sectional design. Corticospinal excitability of several lower limb muscles (i.e., FL, tibialis anterior, and gluteus maximus) was reported in 9 studies on CAI and two studies on ALAS (**Table 2**).

**Table 2:** Summary of included studies.

Study	Participant demographics	Main outcomes	Main results
Kim et al., 2025 (Kim et al., 2025a)	ALAS (*n* = 14; M: 5, F: 9; 19.8 ± 2.0 yrs) HC (*n* = 14; M: 5, F: 9; 20.7 ± 2.3 yrs)	MEP in FL	Higher MEP in FL in ALAS
Kim et al., 2025 (Kim et al., 2025b)	ALAS (*n* = 13; M: 5, F: 8; 19.6 ± 1.9 yrs) HC (*n* = 13; M: 5, F: 8; 20.7 ± 2.4 yrs)	MEP in TA	Higher MEP_100%_ (ES = 1.63, 95%CIs[0.75 to 2.52]), MEP_120%_ (ES = 0.89, [0.08 to 1.69]), and MEP_140%_ (ES = 0.90, [0.09 to 1.71]) in TA in ALAS
McLeod et al., 2015 (McLeod et al., 2015)	CAI (*n* = 21; M: 9, F: 12; 20.81 ± 1.63 yrs) HC (*n* = 24; M: 7, F: 17; 22.54 ± 2.92 yrs)	MEP in FL and vastus medialis	Lower MEP_100%~105%_ in the bilateral FL in CAI
Thompson et al., 2020 (Thompson et al., 2020)	CAI (*n* = 10; M: 3, F: 7; 22.6 ± 2.9 yrs) Coper (*n* = 10; M: 1, F: 9; 23.2 ± 2.6 yrs) HC (*n* = 10; M: 6, F: 4; 22.7 ± 2.3 yrs)	MEP in gluteus maximus, AMT	Higher AMT in gluteus maximus in Coper than in HC (ES = 1.77, [0.31 to 2.96])
Nanbancha et al., 2019 (Nanbancha et al., 2019)	CAI (*n* = 19; M: 15, F: 4; 20.58 ± 1.54 yrs) HC (*n* = 19; M: 15, F: 4; 20.58 ± 1.30 yrs)	MEP, RMT, and latency in TA, FL, and GM	Lower MEP in TA (ES = 0.86, [0.19 to 1.53]) and FL (ES = 1.77, [1.01 to 2.54]) in CAI Longer latency in TA (ES = 0.71, [0.06 to 1.37]) and GM (ES = 0.45, [-0.20 to 1.09]) in CAI
Terada et al., 2022 (Terada et al., 2022)	CAI (*n* = 23; M: 3, F: 20; 23.17 ± 3.54 yrs) Coper (*n* = 23; M: 6, F: 17; 24.74 ± 5.25 yrs) HC (*n* = 24;M: 9, F: 15; 21.08 ± 1.86 yrs)	MEP, CSP, and inhibition in TA	Lower MEP_120%_ in TA in CAI (ES = 0.87) Longer CSP_100%_ in TA in CAI (ES = 0.99, [-0.80 to 0.36])
Terada et al., 2016 (Terada et al., 2016)	CAI (*n* = 16; M: 10, F: 6; 22.19 ± 3.64 yrs) HC (*n* = 17; M: 8, F: 9; 21.24 ± 2.99 yrs)	MEP, CSP, and inhibition in soleus	Higher inhibition in soleus in CAI (ES = 0.82, [0.10 to 1.52])
Pietrosimone et al., 2012 (Pietrosimone and Gribble, 2012)	CAI (*n* = 10; M: 4, F: 6; 21.2 ± 1.23 yrs) HC (*n* = 10; M: 4, F: 6; 21.2 ± 2.3 yrs)	RMT in the bilateral FL	Higher RMT in the injured-side FL (ES = 0.90, [-0.03 to 1.83]) and uninjured-side FL (ES = 0.84, [-0.08 to 1.77]) in CAI
Kosik et al., 2017 (Kosik et al., 2017)	CAI (*n* = 18; M: 4, F: 14; 23.8 ± 3.6 yrs) HC (*n* = 16; M: 6, F: 10; 21.1 ± 2.2 yrs)	MEP and motor cortex’s representation map in FL	Lower map volume (ES = 0.87, [0.15 to 1.55]) and area (ES = 0.71, [0.00 to 1.39]) in FL
Needle et al., 2013 (Needle et al., 2013)	CAI (*n* = 12; M: 6, F: 6; 20.9 ± 4.1 yrs) HC (*n* = 12; M: 6, F: 6; 21.2 ± 2.6 yrs)	AMT in TA, FL, and soleus; CSP in FL	No significant difference
Futatsubashi et al., 2024 (Futatsubashi and Sekiguchi, 2024)	*n* = 43 (M: 43, 82 ankles; 19.8 ± 0.69 yrs) CAI/sub-CAI:32 ankles/20 ankles Coper:17 ankles HC:13 ankles	Threshold in I/O property; CSP in FL	Longer CSP in FL in ankle sprain group (ES = 0.64, [0.14 to 1.14])

F,female; M, male; ALAS, acute lateral ankle sprain; CAI, chronic ankle instability; HC,healthy control; MEP, motor evoked potential; AMT, active motor threshold; CSP, cortical silent period; RMT, resting motor threshold; FL, fibularis longus; TA, tibialis anterior; GM, gastrocnemius medialis; ES, effect size; CIs, confidence intervals.

### 3.4 Alterations in corticospinal excitability in individuals with ankle sprains

Two studies aimed to investigate corticospinal excitability during single-legged standing in individuals with ALAS compared with HCs (Kim et al., 2025a, Kim et al., 2025b). They found that ALAS had high corticospinal excitability at 100% of AMT in the FL (Kim et al., 2025a) and at 100%, 120%, and 140% of AMT in the TA (Kim et al., 2025b).

Nine studies explored the differences in corticospinal excitability in individuals with CAI compared with HC or copers (Pietrosimone and Gribble, 2012; Needle et al., 2013; McLeod et al., 2015; Terada et al., 2016; Kosik et al., 2017; Nanbancha et al., 2019; Thompson et al., 2020; Terada et al., 2022; Futatsubashi and Sekiguchi, 2024). Specifically, three studies evaluated the MEP of the FL and the tibialis anterior (TA) (McLeod et al., 2015; Nanbancha et al., 2019; Terada et al., 2022). Compared with HC, individuals with CAI had lower MEP values in the bilateral FL at 100% and 105% of AMT (McLeod et al., 2015), in the right FL and TA at 120% of RMT (Nanbancha et al., 2019), and in the TA at 120% of AMT (Terada et al., 2022). Pietrosimone et al. found that CAI exhibited higher RMT in the bilateral FL than HCs (Pietrosimone and Gribble, 2012). Furthermore, Nanbancha et al. found that CAI demonstrated prolonged latencies in the TA and gastrocnemius medialis (Nanbancha et al., 2019). Similarly, an extended CSP in TA was observed in CAI by Terada et al (Terada et al., 2022). By measuring the MEP and CSP of the TA and soleus, they assessed corticospinal inhibition by calculating the ratio of CSP to MEP at 120% of AMT and found that the CSP/MEP ratio was higher in individuals with CAI than in HCs, indicating increased corticospinal inhibition in the soleus (Terada et al., 2016). Thompson et al. used TMS to assess the AMT of the gluteus maximus and found that the AMT of the gluteus maximus in the copers was higher than that in HCs (Thompson et al., 2020). Several studies also calculated other secondary outcomes of TMS-derived recruitment curves to measure cortical excitability. CAI exhibited smaller volumes and areas in the motor cortex’s representation map of the FL compared with HCs (Kosik et al., 2017). Futatsubashi et al. further found that the threshold of the input–output (I/O) property of CAI was higher than that of HCs, and the duration of the silent period in the subsequent ankle sprain group was significantly extended (Futatsubashi and Sekiguchi, 2024). Nonetheless, regarding primary outcomes, they found no differences in MEP for vastus medialis (McLeod et al., 2015). Only one study reported no significant differences in AMT, slope, and intensity at the 50% peak slope of the recruitment curves of the TA, FL, and soleus between the two groups (Needle et al., 2013). Besides, effect sizes and associated 95% confidence intervals of the main outcomes in the included studies were described in the **Supplementary Figure 1**.

## 4 Discussion

TMS is a widely used technique for detecting corticospinal excitability from the cerebral cortex to the target muscles. It achieves this by administering precise magnetic pulses to the motor cortex and recording the MEP in the target muscles, thereby providing feedback on whether the pathways are damaged (Klomjai et al., 2015; Derosiere et al., 2020). The included studies indicate that, compared with HCs or copers, individuals with CAI exhibit lower MEP values in the FL and TA, suggesting abnormalities in the neural pathways for lower limb neuromuscular control in CAI (McLeod et al., 2015; Nanbancha et al., 2019; Terada et al., 2022). One possible explanation is that injury to the ankle joint ligaments may reduce sensory nerve input, preventing the central nervous system from effectively integrating this information and resulting in delayed muscle movement control. Additionally, the prolonged obstruction of sensory information transmission to the central nervous system may correspond to potential changes in the central nervous system (Hertel, 2008). The reduced excitability of the corticospinal pathways in the lower limb muscles of the CAI population may also increase the risk of ankle joint re-injury (Del Olmo et al., 2006).

By contrast, individuals with ALAS showed higher MEP in the FL and soleus, suggesting an enhancement in corticospinal excitability of the lower limb muscles following an acute ankle sprain. The observed increase may reflect a compensatory neural response by increasing the activity of excitatory neurons to maintain postural balance and prevent further re-injury (Kim et al., 2025a, Kim et al., 2025b). Furthermore, acute injuries can cause significant changes in the neurophysiological state, including abnormal sensory input, disordered output control, increased pain sensation, limited mobility, emotional stress, fear of re-injury, and increased cognitive load. Thus, ALAS must be allocated increased attention because it produces heightened motor output signals during movement tasks, thereby leading to enhanced corticospinal excitability (Miao et al., 2017).

Compared with the HC, the AMT of the gluteus maximus in copers was higher. AMT is regarded as a measurement marker for evaluating the membrane excitability of pyramidal neurons. A higher AMT indicates a decrease in the excitability of the corticospinal system, indicating that a strong stimulus is required to activate the corresponding cortical neurons. Proper postural control requires the corticospinal system to accurately transmit movement information to the target muscles, thereby facilitating accurate strength recruitment during postural control (Riemann and Lephart, 2002). A previous study showed that postural control in the coper group is superior to that of the CAI group, suggesting that they may successfully reorganize corticospinal activity after ankle joint injury, thereby forming an optimal proximal posture control strategy. The coper achieves movement by enhancing the function of the distal muscles of the ankle joint, avoiding chronic functional disorders after injury (Thompson et al., 2020).

Lower corticospinal excitability may be partly associated with the greater corticospinal inhibition. Specifically, CSP mainly reflects the inhibitory activity of the cortex, illustrating the inhibitory function of the motor cortex (Terada et al., 2016, Terada et al., 2022). The latency period denotes a delay in the neural regulation pathway from the corticospinal tract to the target muscle (Nanbancha et al., 2019). The prolonged CSP and latency period in CAI indicate an enhancement of corticospinal inhibition. Specifically, the extended CSP in the TA and the high CSP/MEP ratio in the soleus of CAI, as well as the prolonged latency periods of the TA and FL, reflect the adaptive mechanisms of the central nervous system in response to peripheral musculoskeletal injuries. The enhancement of inhibitory control affects abnormal corticospinal excitability, responding to reduced motor control and an increased risk of re-injury (Terada et al., 2016; Nanbancha et al., 2019; Terada et al., 2022). In addition, the RMT values of the bilateral FL in CAI are high, indicating a decline in the individual’s ability to independently regulate the target muscles, thereby increasing the likelihood of recurrent ankle sprains (Pietrosimone and Gribble, 2012). Notably, CSP is commonly interpreted as an index of cortical inhibition, it is influenced by multiple physiological and methodological factors. Thus, appropriate spinal-reflex measures are available to further directly establish changes in spinal segmental activity.

Corticospinal excitability and the inhibitory functions of the lower limb-related muscles in ALAS and CAI change over time, suggesting underlying abnormalities in the corticospinal pathway among individuals with musculoskeletal injuries. The primary abnormalities are associated with poor neuromuscular control performance. Therefore, the effect of ankle sprains on corticospinal excitability should be investigated to help clarify the neurological mechanisms underlying functional dysfunctions of the ankle for clinical rehabilitation.

Peripheral foot and ankle dysfunction in CAI mainly includes decreased proprioception, impaired neuromuscular control, weak muscle strength, abnormal muscle activation patterns, and impaired postural control (Xiao et al., 2024; Liang et al., 2026). Normal regulation of the central nervous system is essential for sustaining peripheral functions. Therefore, the reduced performance of foot and ankle functions is associated with decreased corticospinal excitability in CAI. Moreover, reductions in corticospinal excitability may associate with delayed or insufficient muscle activation, thereby affecting stability and movement control. Previous studies have found a significant negative correlation between the MEP of the soleus and FAAM-ADL (Harkey et al., 2016), a negative correlation between the CSP of the TA and DFRoM in individuals with CAI (Kosik et al., 2021), and a negative correlation between the RMT of the FL and FADI (Pietrosimone and Gribble, 2012). These findings indicate that changes in corticospinal excitability can directly affect peripheral function, possibly resulting in recurrent injury. Compared with HCs, individuals with CAI exhibit an increased threshold for the corticospinal excitatory input/output characteristics of the FL due to repeated ankle sprains. Besides, Xue et al. found that the LAS mouse model exhibited maladaptive alterations in the corticospinal tract (CST), and further proved that a genetic predisposition to ankle sprains associated with reduced fractional anisotropy and increased orientation dispersion index of the CST (Xue et al., 2023b, Xue et al., 2025). These regulatory changes may associate with deficiencies in ankle joint control function, increasing the risk of recurrent ankle sprains (Futatsubashi and Sekiguchi, 2024). Furthermore, several studies used functional magnetic resonance imaging to explore brain activity in the cerebral cortex and found that CAI exhibits aberrant changes in intrinsic activity within the sensorimotor network under resting-state and task-related conditions (Shen et al., 2022; Xiao et al., 2025). Notably, the joint position sense is remarkably associated with task-related cortical activation in the left precentral gyrus, bilateral putamen, and supplementary motor area in athletes with CAI but not in HCs (Xiao et al., 2025). CAI may encounter difficulties in coordinating movements, particularly for ankle control and stabilization, because of low cortical activation in specific brain regions.

The peripheral foot and ankle dysfunction in CAI is exhibited in multiple aspects. Changes in the excitability of the spinal cord and supraspinal elements may result in limited foot and ankle function during daily activities and movements in individuals with CAI. Therefore, future research on CAI rehabilitation should focus on improving peripheral foot and ankle function and correcting the abnormal changes in corticospinal excitability. Moreover, TMS can be used for rehabilitation assessment and treatment.

### 4.1 Implications

Our findings suggest that the TMS technique should be employed not merely as a prognostic screening tool at the acute stage, but also as a longitudinal monitoring tool in chronic stages, particularly using MEP and CSP to assess the neurophysiological changes for determining therapeutic after-effects or natural recovery trajectories. Besides, summarizing corticospinal excitability in ankle sprains offers significant theoretical insights for the development of rehabilitation protocols. Currently, traditional rehabilitation strategies use functional training to enhance peripheral sensorimotor functions, such as balance training and strength training (Guo et al., 2024; Park et al., 2024). Functional training can influence the central nervous system to some extent, but direct rehabilitation strategies at the spinal cord level (e.g., spinal cord electrical stimulation) and even at the cerebral cortex level (e.g., transcranial magnetic stimulation, transcranial electrical stimulation, and transcranial ultrasound stimulation) can effectively address the abnormal corticospinal pathways in CAI (Bruce et al., 2020; Xiao et al., 2026). Therefore, future studies should confirm whether the intervention that regulates the excitability of the spinal cord and cerebral cortex can effectively reconfigure the normal neuromuscular control pathways and the impaired peripheral sensorimotor functions (**Figure 2**).

**Figure 2:**
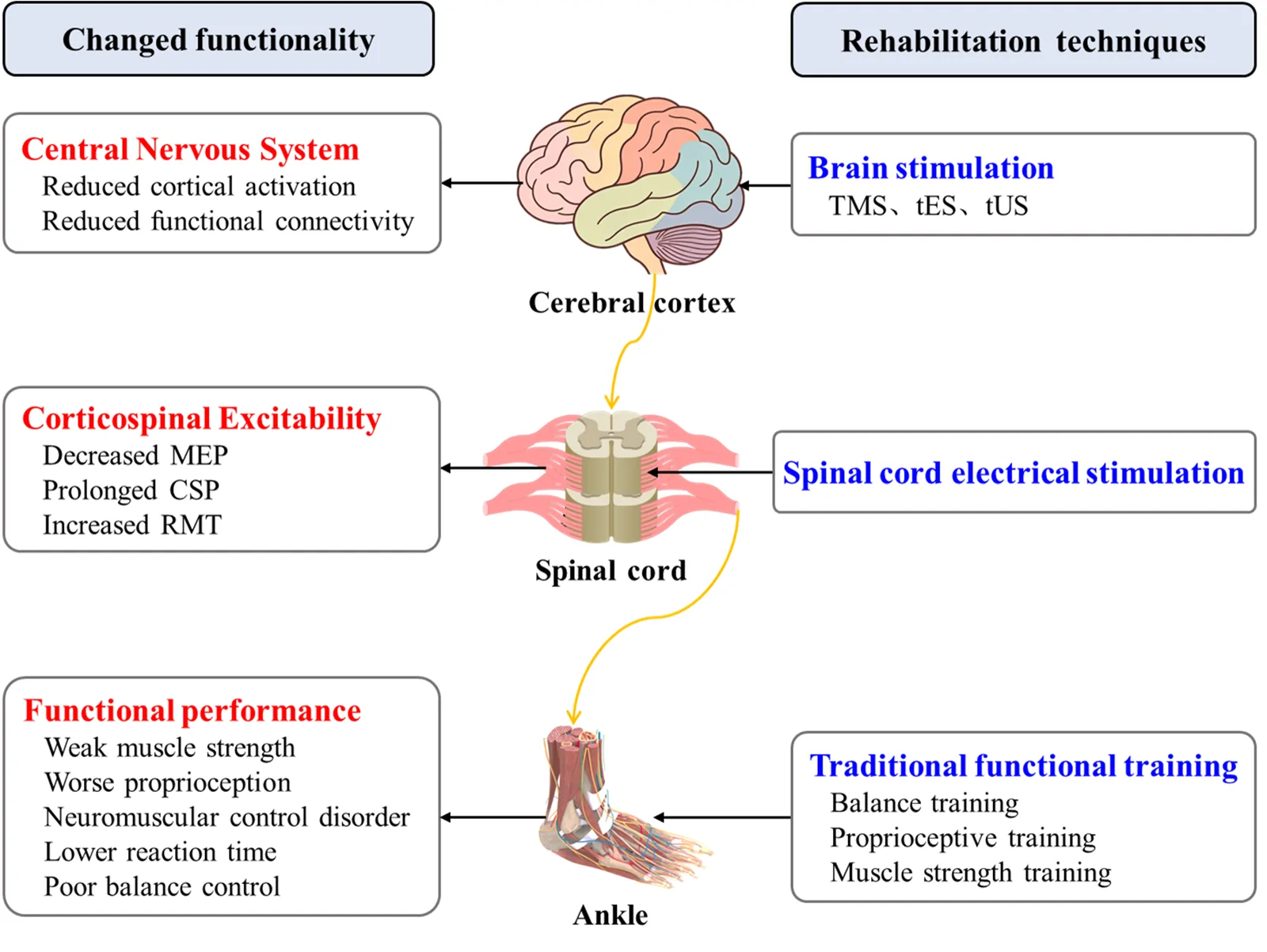
Simplified diagram of changed functionality and main rehabilitation techniques for individuals with CAI. MEP, motor evoked potential; CSP, cortical silent period; RMT, resting motor threshold; TMS, transcranial magnetic stimulation; tES:transcranial electrical stimulation; tUS, transcranial ultrasound stimulation.

### 4.2 Limitations

The results of this systematic review should be interpreted with caution because of the methodological limitations present in the included studies. First, this review protocol was not prospectively registered. Second, the included studies were all cross-sectional studies, so the underlying mechanism in corticospinal excitability after ankle sprains and the causal relationship between ankle sprains and changed functionality remain unknown. The current findings can only provide a certain basis for the understanding of the underlying mechanism of CAI. Future studies may implement a long-term follow-up design to explore the causal relationship between changes in central nervous system function and peripheral functional dysfunctions in an animal model of ankle sprain. Third, all included studies had high heterogeneity in the primary outcomes (e.g. MEP, CSP, and AMT), target muscles, TMS protocols, and stimulation intensities. Thus, conducting a meta-analysis is necessary in future studies by focusing on a key outcome in the same target muscles. Fourth, non-English studies were excluded. Thus, we clearly state that this may limit the generalizability of our findings to non-English-speaking regions, and we recommend that future systematic reviews attempt to include a broader language scope to further validate our findings. Finally, many factors are related to musculoskeletal injuries, such as age, gender, exercise habits, and exercise ability, and these factors are also associated with the function of the central nervous system. Therefore, the influence of these confounding factors on ankle sprains must be elucidated in the future.

## 5 Conclusion

Individuals with ankle sprains exhibit underlying abnormalities in the neurophysiological characteristics of neuromuscular control. Individuals with CAI exhibit a lower corticospinal excitability (i.e., diminished MEP) and higher cortical inhibitory activity (i.e., prolonged CSP). Individuals with acute ankle sprain exhibit higher corticospinal excitability based on only two studies, which should be presented cautiously as a preliminary finding requiring further confirmation. These findings indicate that acute or recurrent ankle sprains are associated with potential changes in the central nervous system, suggesting that altered corticospinal excitability may associate with the development or persistence of CAI after ALAS. Future studies should investigate whether restoring impaired peripheral sensorimotor functions can reestablish normal neuromuscular control pathways.

## Data Availability

The original contributions presented in the study are included in the article/**Supplementary Material**, further inquiries can be directed to the corresponding author/s.
