# Supplementary material for: Alterations in corticospinal excitability in individuals with ankle sprains: a systematic review of transcranial magnetic stimulation studies
Source: Front Physiol. 2026 Sep 21;17:1951700. doi: 10.3389/fphys.2026.1951700 (PMC13634940; doi:10.3389/fphys.2026.1951700)
Supplement: Supplementary file 1 [file Supplementaryfile1.docx]

**Supplementary Figure 1.** Effect sizes and associated 95% confidence intervals of the main outcomes in the included studies. CAI: chronic ankle instability; MEP: motor evoked potential; AMT: active motor threshold; CSP: cortical silent period; RMT: resting motor threshold; FL: fibularis longus; TA: tibialis anterior; GM: gastrocnemius medialis.
